# SpaVerb-WN—A megastudy of naming times for 4562 Spanish verbs: Effects of psycholinguistic and motor content variables

**DOI:** 10.3758/s13428-021-01734-y

**Published:** 2021-12-16

**Authors:** Romina San Miguel-Abella, Miguel Ángel Pérez-Sánchez, Fernando Cuetos, Javier Marín, María González-Nosti

**Affiliations:** 1 Departamento de Psicología, Universidad de Oviedo, Plaza de Feijoo, s/n, 33003 Oviedo, Asturias Spain; 2 Departamento de Psicología Básica y Metodología, Universidad de Murcia, Murcia, Spain

**Keywords:** Word naming, Reaction times, Motor content, Grammatical class, Verbs, Megastudy

## Abstract

Several studies have been carried out in various languages to explore the role of the main psycholinguistic variables in word naming, mainly in nouns. However, reading of verbs has not been explored to the same extent, despite the differences that have been found between the processing of nouns and verbs. To reduce this research gap, we present here SpaVerb-WN, a megastudy of word naming in Spanish, with response times (RT) for 4562 verbs. RT were obtained from at least 20 healthy adult participants in a reading-aloud task. Several research questions on the role of syllable frequency, word length, neighbourhood, frequency, age of acquisition (AoA), and the novel variable ‘motor content’ in verb naming were also examined. Linear mixed-effects model analyses indicated that (1) RT increase in with increasing word length and with decreasing neighbourhood size, (2) syllable frequency does not show a significant effect on RT, (3) AoA mediates the effect of motor content, with a positive slope of motor content at low AoA scores and a negative slope at high AoA scores, and (4) there is an interaction between word frequency and AoA, in which the AoA effect for low-frequency verbs gradually decreases as frequency increases. The results are discussed in relation to existing evidence and in the context of the consistency of the spelling–sound mappings in Spanish.

## Introduction

Reading is a skill that consists of transforming graphic signs into sounds, in the case of reading aloud, or directly into meanings, in the case of reading comprehension. Since the end of the last century, research has shown that psycholinguistic characteristics of the stimuli influence reading, although some differential effects between languages have been found. Importantly, the largest differences in the effects of these variables have been found between languages that vary in the transparency of their spelling and in their metric systems (Ardila, 1991; Ardila & Cuetos, 2016). Most studies on reading have been carried out in English, and consequently many of the prominent models of word recognition and reading are based on the results from the available studies in this language (for a review, see Norris, 2013). However, English has several linguistic characteristics that could critically influence word processing and that are not shared by other languages. For instance, English has opaque spelling, with highly inconsistent grapheme–phoneme correspondence, no consistent accent assignment, and unclear syllabic segmentation. Spanish represents a suitable language for comparison because it has transparent spelling, high consistency in grapheme–phoneme correspondence, consistent accent rules, and clear syllabic limits. Importantly, as we will summarize next, evidence shows that the effects on word processing of several psycholinguistic variables associated with the above features vary from one language to another. Therefore, the effects on word processing found in one language might not be directly extrapolated to others, and consequently, English-based models of reading would need to be adapted to other languages taking into account the existence and the extent of the involvement of the psycholinguistic variables mentioned above.

Lexical frequency is one such variable that has been consistently shown to have a prominent influence on word reading. High-frequency words require less time to be named than low-frequency words in different languages (for a review, see Ghyselinck et al., 2004), although this variable is more influential in English, a language with deep spelling (Forster & Chambers, 1973; Rubenstein et al., 1971), than in Spanish, which has a transparent orthography (Davies et al., 2013, 2014; but see Cuetos & Barbón, 2006). Word length has a direct effect on reading speed in Spanish, with less time devoted to the reading of short words as compared to long words (Álvarez et al., 1999; Cuetos et al., 1997; Davies et al., 2013), while evidence suggests that in English, the relationship between word length and naming times may be a quadratic function or a U-shaped form instead of a linear one (Yap & Balota, 2009; see Barton et al., 2014 for a review). Orthographic neighbourhoods of words have also been found to produce an effect on word naming, with words with larger neighbourhoods taking less time to read aloud. This effect has been consistently found across opaque (e.g., Andrews, 1997) and transparent languages (e.g., Barca et al., 2002; Davies et al., 2013). Age of acquisition (AoA) is a decisive variable in word naming in English (e.g., Brysbaert & Cortese, 2011; Cortese & Khanna, 2007; Cortese & Schock, 2013; see Juhasz, 2005; Johnston & Barry, 2006, for reviews) and in Portuguese (a language with an intermediate-depth spelling; Soares et al., 2019), with early-acquired words read aloud faster than late-acquired ones. However, AoA has been found to produce inconsistent effects in transparent languages, with mostly null effects in Italian (e.g., Barca et al., 2002; Burani et al., 2007; Davies et al., 2014; but see Bates et al., 2001, for positive effects), mixed results in Spanish (see Cuetos & Barbón, 2006, and Davies et al., 2013, for positive effects, and Alonso et al., 2015, 2016, and Davies et al., 2014, for null effects), and a positive effect on naming times in Turkish (Raman, 2006, 2018). These findings seem to be explained by two factors: the consistency of spelling–sound mappings in each language, where more inconsistent mappings are associated with a larger effect of AoA, and the involvement of semantics in the reading task, with a higher effect of AoA in tasks that require a more intense semantic processing of the input. Evidence of the importance of the consistency of spelling–sound mappings can be found in a study by Wilson et al. (2012), who reported an AoA effect for Italian words with irregular stress (i.e., inconsistent mapping), but not for words with regular stress (i.e., consistent mapping). The evidence of the involvement of semantics was first provided by Wilson et al. (2013), who observed that AoA affected reading times in Spanish only when participants named highly imageable words (i.e., high semantic involvement) and not when they responded to words with a wider range of imageability values (i.e., lower semantic involvement). Furthermore, Davies et al. (2014, experiment 1) reanalysed the results obtained by Cuetos and Barbón (2006) considering the raw variables corresponding to the attributes of the stimuli in order to examine specifically the role of imageability of words as a modulator of the AoA effect. Davies et al. (2014, experiment 2) also performed a virtual experiment (the naming times were taken from Davies et al., 2013) with 626 words that were centred in the imageability scores. In both experiments, results were compared with those obtained by Barca et al. (2002) in Italian for the same subset of words. In experiment 1, Davies et al. (2014) observed that the AoA effect was significant in Spanish but not in Italian when the imageability scores were included in the analysis. By contrast, in experiment 2, they found null AoA effects in both Spanish and Italian. Davies et al. (2014) concluded, as Wilson et al. (2013) had previously suggested, that the significant effect of AoA reported by Cuetos and Barbón (2006) could be due to the highly imaginable words employed in that study.

Additionally, the interaction between AoA and lexical frequency, when examined in word naming, has shown differences across languages. An interaction such that the AoA effect only occurs or is larger in low-frequency words has been encountered in English (Catling & Elsherif, 2020; Cortese & Schock, 2013; Cortese et al., 2018; Dirix & Duyck, 2017; but see Juhasz & Rayner, 2006), while in Italian and Spanish the interaction has not been observed (Burani et al., 2007; Wilson et al., 2013). These results suggest that frequency and AoA may exert different influences at distinct stages of word reading depending on the language transparency. Regarding syllable frequency, the seminal work by Carreiras et al. (1993) was the first to demonstrate a first-syllable frequency effect in word naming, with words that start with high-frequency syllables being read faster than those that start with low-frequency syllables (see also Carreiras et al., 2006; Carreiras & Perea, 2004). The effect has been replicated in other languages, such as English (Macizo & Van Petten, 2006) and Korean (Simpson & Kang, 2004). This facilitatory effect was explained by the fact that syllable frequency plays a role in post-lexical access in speech production, more specifically at the phonetic encoding stage (i.e., when accessing stored syllabic units), with faster access to articulatory-phonetic syllable programs for higher-frequency syllables (e.g., Levelt et al., 1999). However, Conrad et al. (2006) found an inhibitory effect in German. They suggested that the differential effects arise from the differences in stress ambiguity between languages. For instance, in Spanish, syllable structure can be inferred via superficial orthographic analyses, and therefore syllabic units can be activated earlier and independently of lexical access in processing, leading to faster motor responses for words with high first-syllable frequency. By contrast, in German a prelexical or sublexical facilitation of motor production by syllable structure is not possible, because lexical access is required to complete phonological information about the word, including the stressed syllable. Finally, the grammar category of words has also been found to influence performance, with open-class words taking longer to read than closed-class words, which may also be because closed-class words tend to be high-frequency and short-length words (Ellis et al., 1983).

These results show, on the one hand, that the effects of the above variables on word reading vary from one language to another and, more specifically, that the effects found in a language with opaque spelling, such as English, should not be directly transferred to languages with transparent spelling, such as Spanish, and vice versa. On the other hand, findings from previous studies suggest the need for further research with a more specific focus on the spelling systems of different languages. In light of this research gap, we present a megastudy which employs a large amount of word naming data in Spanish and analyses the effects of different psycholinguistic variables on the given task.

In the study of variables, most of the research on reading has used a factorial methodology, in which several variables of interest are manipulated between groups of items while the rest of the variables remain constant. An important disadvantage or limitation of this method is the difficulty in simultaneously exploring multiple variables. Moreover, selecting material under too many constraints can lead to the need to reduce the number of items, which in turns results in reduced statistical power, or in the inclusion of bizarre words, with negative consequences for the generalizability of the findings. By contrast, several recent megastudies have collected behavioural data for thousands of items (commonly an extensive sample of words that robustly represents the whole lexicon of a language). Then, a large number of psycholinguistic variables are taken to explore which of them contribute to explaining the behavioural data (commonly by regression analyses). Nowadays, there is no doubt that megastudies have methodologically and theoretically contributed to the scientific progress in cognitive psychology, and especially in psycholinguistics (see Balota et al., 2012; Keuleers & Balota, 2015; Kuperman, 2015, for the advantages of megastudies).

Although the term ‘megastudy’ was not coined until 1989 in the paper by Seidenberg and Waters (1989), in which the reading times of almost 3000 words were collected, the tradition of megastudies in the English language dates back a few years earlier. The first study that can be considered a megastudy was carried out by Coltheart (1981), with almost 100,000 words. Later studies included languages other than English, such as German and Dutch, and a few years later the iconic English Lexicon Project was published (Balota et al., 2007), with chronometric data and psycholinguistic variable norms for more than 40,000 words. The first megastudy in the Spanish language is much more recent. It was conducted by Davies et al. (2013), who obtained the reading times for 2764 monosyllabic and multisyllabic words belonging to different grammatical classes (nouns, verbs, and adjectives) in a word naming task. In addition to the chronometric data, values of lexical and bigram frequency, AoA, length, imageability, familiarity, and number of neighbours were included in a regression analysis. The results showed the influence of both orthographic form (i.e., length, bigram type frequency) and lexical (frequency, familiarity, and AoA) and semantic factors (i.e., AoA and imageability). According to the authors, the results confirmed that word reading in Spanish is realized through spelling–sound mappings involving lexical and sublexical units. Moreover, they highlighted that an effect of semantic knowledge was observed even for words with regular spelling–sound correspondence.

A second megastudy in Spanish was conducted by González-Nosti et al. (2014), who employed the same set of 2765 words used by Davies et al. (2013) to examine the influence of psycholinguistic variables on word recognition latencies in a lexical decision task. The results showed that frequency and AoA had significant effects on reaction times regardless of the type of words used, while length, orthographic neighbourhood, and imageability were significant only in specific groups of words. The authors concluded that reading in Spanish, as in deep orthography languages, also depends on a combination of lexical and sublexical strategies.

The most recent megastudy carried out in Spanish is the SPALEX project (Aguasvivas et al., 2018), in which a lexical decision task was employed with 169,628 Spanish native speakers from Spain and Latin America. The task was executed on a website where participants responded with no time limit. SPALEX, which represents the largest database in Spanish available to date (including 45,389 words and 56,855 non-words), has been used to examine the influence on reading of such socio-demographic variables as gender, age, country of origin, and level of education (Aguasvivas et al., 2020). However, SPALEX does not provide a comprehensive analysis of the psycholinguistic variables involved in word recognition.

In contrast to the majority of factorial studies in Spanish that have been carried out only with nouns, the above-mentioned megastudies included other grammatical categories, such as verbs and adjectives. However, knowledge about the influence of the grammatical category of words in reading is still scarce. Davies et al. (2013) only provided evidence that verbs and nouns were read significantly more slowly than adjectives. González-Nosti et al. (2014) did not include the grammatical category factor in the analysis, and to date there are no studies based on the SPALEX project (Aguasvivas et al., 2018) that have explored the role of this factor in word recognition. Because of the lack of direct evidence, the question naturally arises as to whether the results obtained with nouns may be directly generalized to words of other grammatical categories. However, some insights can be obtained from neuropsychological studies, which provide evidence of dissociations in object/action naming and comprehension of motor/abstract verbs.

Taking into account that the distinction between nouns and verbs is not strictly the same as that between objects and actions (Vigliocco et al., 2011), several transcranial magnetic stimulation and neuroimaging studies have clearly shown a dissociation between object and action word processing in the brain: the left temporal cortex is associated with the naming of objects (e.g., shoe), whereas the left prefrontal cortex is associated with action naming (e.g., running) (Cappa, 2008; Damasio & Tranel, 1993; Martin, 2007; Martin & Chao, 2001; Tranel et al., 1997). Brass et al. (2007) showed that brain responses for comprehension of verbs with motor meanings (e.g., to run) differ from those for verbs with abstract meanings (e.g., to think). Riccardi et al. (2019) found that localized damage in the left anterior middle temporal gyrus significantly impaired comprehension of more abstract verbs to a greater extent as compared to action verbs. This dissociation supports the idea that semantic processing of action and abstract verbs relies on partially separate brain networks, with action verbs producing greater activation in the sensorimotor cortex. These findings, obtained mainly from data on patients with brain injury and from studies carried out in healthy people using functional magnetic resonance imaging (fMRI), have provided important support for the assertions of the *embodiment theory*. According to Glenberg (2015), this theory holds that cognition is formed through sensorimotor experience, and thus both our behaviour and other processes that we would label as abstract, such as language, are actually controlled by systems of perception, action, and emotion that interact with the environment. In the case of verb processing, this theory states that semantic content related to actions is represented in a more sensorial form, and therefore verbs that involve movements are associated with sensorimotor neural networks. These networks include areas of both motor and sensory systems, showing a correspondence between the frontal regions involved in the processing of action words and the motor areas that allow the performance of action and movement. The embodiment theory has also been used in recent years to explain the deficit in the processing of language with high motor content in people with some type of motor impairment disease, such as certain types of aphasia, Parkinson's disease (PD), or primary cervical dystonia (PCD) (Aziz-Zadeh et al., 2006; Boulenger et al., 2009; Hauk et al., 2004; Rizzolatti et al., 2002). For example, it has been observed that action-related lexical-semantic impairment is characteristic of PD, so there are marked deficits in the processing of verbs with high motor content and of some nouns that involve movement as compared to abstract verbs and other nouns (Bocanegra et al., 2017; Fernandino et al., 2013; Herrera et al., 2012). In addition, better performance was found in a word association task related to the administration of a precursor of dopamine (i.e., Levodopa), which was used as medical treatment to improve motor symptoms in PD patients (Herrera & Cuetos, 2013). This finding suggests that the function of the lexical-semantic system may be disrupted in the absence of dopamine. Nevertheless, Bayram and Akbostanci (2018) do not interpret the slight action deficit in PD and PCD as an outcome of a strong or pure embodied representation of high-motor-content verbs, as such deficit may be compensated by using non-action verbs. This is in line with Mahon and Caramazza’s (2008) intermediate position between ‘embodied’ and ‘disembodied’ cognition, in which sensory and motor information play an important, but non-essential, supportive role in action processing.

Megastudies with verbs offer the opportunity to verify whether the processing of verbs diverges from the processing of nouns in healthy individuals. To date, there are no studies carried out exclusively with verbs, so it is unknown which psycholinguistic variables affect the processing of this grammatical category and to what extent. Moreover, the inclusion in the analysis of variables specifically related to verbs, such as ‘motor content’, may help to clarify why some patients with motor deficits (e.g., PD and PCD) have difficulties processing verbs with high motor content. The motor content of an action word refers to the amount of mobility it alludes to, understood as the amount of displacement and/or movement of the different parts of the body (fingers, hands, legs, etc.) involved in its execution. Motor content is obtained by averaging the ratings of adult participants on a scale, where the lowest scores correspond to verbs that imply very little movement (e.g., to vegetate), and the highest scores are linked to verbs that involve a great deal of movement (e.g., to train) (San Miguel Abella & González-Nosti, 2020). The existence of a published database in Spanish (San Miguel Abella & González-Nosti, 2020) with the norms on this variable facilitates the achievement of the aim of the current study.

Aside from theoretical questions about verb and noun processing, this broad base of naming latencies is inherently interesting, as it includes norms for both lexical and sublexical variables that may facilitate the stimulus selection process for other investigators. The availability of both individual and average RT is also another valuable resource for researchers working in fields as diverse as language processing, attention, or memory, or even in the creation of computer models of word recognition. At the clinical level, this database would also have application particularly in the selection of materials for rehabilitation, since RT allow us to test which words are recognized more quickly and therefore may be easier for patients with language impairment.

Therefore, in this context, the main objective of this megastudy is to provide a behavioural database of word reading accuracy and speed data from healthy adults for a wide range of Spanish verbs. The second objective is to explore different phonetic, sublexical, lexical, and lexical-semantic variables that may be involved in the reading of given words. Furthermore, we were interested in investigating the influence of the motor content of verbs, as well as the interactions between this variable and each of the psycholinguistic variables selected. To this end, we used a word naming task which consisted in showing a word on a computer screen to participants, who named it aloud as quickly and accurately as possible (Balota et al., 2007). Then, we performed several analyses using linear mixed-effects models (LMM) to address whether the classical variables of word frequency, AoA, length, neighbourhood size, and syllable frequency, the novel variable of motor content, and more specifically the interaction between AoA and word frequency, affect verb naming latencies.

## Method

### Participants

A total of 126 native speakers of Spanish participated in this study. Their mean age was 22.0 years (range: 19–33; *SD*: 2.5), and 100 of them (74%) were women. They were all undergraduate, master’s, or doctoral students at the Faculty of Psychology of the University of Oviedo (Spain). The participants took part in this experiment in exchange for academic credit or as volunteers. They all had normal or normal corrected vision and no reading, speech, or neurological disorders when they performed the task. The sample size for this study was selected considering those used in other studies that collected RT for a similar number of items, which have reported 20 responses per item, and we also followed the recommendations of Brysbaert and Stevens (2018) for registering a minimum of 1600 observations per condition in designs with repeated measures.

### Materials

We used a set of 4562 Spanish verbs in the infinitive or pronominal (i.e., infinitive + the reflexive pronoun ‘-se’) forms taken from San Miguel Abella and González-Nosti's (2020) motor content database. The entire set of terms also had previous norms for AoA (Alonso et al., 2016). Word length, frequency, and phonological and orthographic neighbourhood were collected from the EsPal database (Duchon et al., 2013). First-syllable frequency was estimated by the syllabification of the CREA corpus (Real Academia Española, 2008). In its current state, this lexical database is composed of about 170 million Spanish words, with the majority of those (>90%) coming from written records. The syllabification process was performed by programming in Visual Basic for Applications language the algorithm proposed by Cuayáhuitl (2004). Then, the ‘type’ first-syllable frequencies were obtained by counting, in the whole corpus, the number of different words starting with the given syllable, and the ‘token’ first-syllable frequencies were obtained by the sum of the frequencies of the words starting with the given syllable.

The selected verbs were randomly divided into nine blocks with 503 or 504 words each, which were distributed among the different participants. We needed to add one more block to register 33 verbs that were not properly presented in other blocks because of a failure in the run scripts. This ‘rescue’ block also included 469 verbs randomly selected from the whole set, which were used as fillers in order to maintain the same conditions as in the rest of blocks. We also used four additional verbs to be presented as warming trials at the beginning of the task.

### Procedure

The word reading task was performed in soundproof booths at the lab of the Faculty of Psychology at the University of Oviedo. Item presentation and response registering were done by means of DMDX software (Forster & Forster, 2003). The data were collected during individual sessions. Each participant first filled out and signed the informed consent and then performed the task. The participants were presented with the verbs one by one on a computer screen (15.6 inch) in lowercase letters, font Arial 11-point, and black characters on white background. They were asked to read the verbs aloud as fast and accurately as possible. They sat approximately 60 cm from the monitor and were given a headset with a high-sensitivity microphone connected to the computer.

The sequence of events in each experimental trial started with an asterisk as a fixation point in the centre of the screen for 500 ms. Immediately after that, a word appeared, which remained on the screen until the participant initiated the verbal response or until 1500 ms. Four warming trials were presented at the beginning of each block. Then, each participant was presented with the target items in randomized order, with breaks every 50 stimuli in which each participant decided on their duration. The average duration of the task was around 25 minutes. Forty-five participants completed one block, 77 two blocks, and four completed three blocks of items. Each subject performed a maximum of one script per day. Each block was presented to either 20 or 21 participants.

## Results

We first report the data trimming for analysis, removing response errors and outliers. We then report some basic statistics for the response times obtained and for the characteristics of the psycholinguistic variables. Next we present the pairwise correlation matrix of variables and the steps taken to avoid the potential problem of multicollinearity. Finally, we report the results from several LMM analyses carried out on the word naming times.

### Data trimming

A total of 105,651 responses were obtained from 126 participants to the 4562 verbs^1^. Each response was coded as correct or incorrect using the CheckVocal program (Protopapas, 2007). As a result, a total of 146 omissions and 2713 mispronunciations (representing 0.14% and 2.56% of the total responses, respectively) were removed from the data set. Regarding the correct responses (*M* = 97.28%, *SD* = 5.74, min = 9.52%, max = 100%), 4319 verbs (94.7% of the set) were correctly read by at least 90% of participants and only six verbs (0.1% of the set) were correctly named by less than 50% of participants. Then, 2436 responses with extremely fast (RT < 200 ms or 2 *SD* below the participant’s mean) or slow (RT 2 *SD* above the participant’s mean) latencies were also excluded from the data set, which represented 2.37% of the total correct responses. After the data trimming, a data set containing 100,356 naming latencies was available for the next analyses. A file with the data at trial level and another with the accuracy scores and mean latencies of correct responses for each word are available as supplementary material to this paper.

### Descriptive statistics

**Fig. 1:**
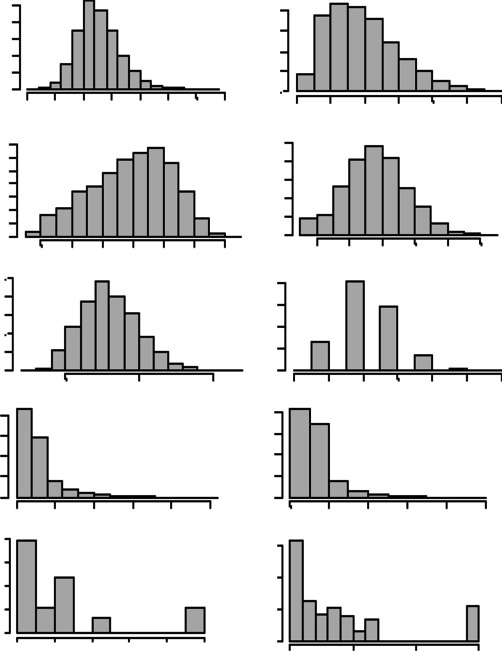
Histograms of each of the variables

Descriptive statistics and histograms of the distribution of the RT means and the variables’ scores by item are presented in Table 1 and Fig. 1, respectively. Lexical frequency values were transformed into Zipf scores (log10(fpmw)+3; van Heuven et al., 2014), and this was the metric used in all subsequent analyses. As can be seen in Fig. 1, the distribution of RT values fitted a normal curve but had the typical moderate positive bias of this outcome (skewness = 0.660). Both orthographic and phonological neighbourhood sizes were highly skewed (skewness of 2.334 and 2.549, respectively), and motor content and number of letters and syllables showed moderate-to-low positive skews (skewness of 0.715, 0.430, and 0.416, respectively). By contrast, AoA had a negative low skew (−0.315), and Zipf showed no skewness (0.019). Regarding the mean and range of the predictor variables, motor content showed scores that covered the entirety of the corresponding 1-to-7 scale, with a mean (over 2.9 points) under the midpoint of the scale (i.e. 4 points); AoA scores covered a range of 13 years, with a mean age near 10 years; and word length and neighbourhood measures showed representative means and ranges of the whole Spanish lexicon (i.e., written and web corpus of EsPal: letters, *M* = 9.2, min = 1, max = 23; syllables, *M* = 3.8, min = 1, max = 10; orthographic neighbourhood, *M* = 2.2, min = 0, max = 45; and phonological neighbourhood, *M* = 4.7, min = 0, max = 117; see Duchon et al., 2013). Zipf deserves a special mention: mean and median Zipf frequency values (over 2.7 points) show that our words are mostly low-to-mid-frequency words as classified according to the intuitive scale suggested by van Heuven et al. (2014) for this variable (i.e., values of 1–3 represent low-frequency words, and values of 4–7 represent high-frequency words). However, it must be noted that there are also a considerable number of high-frequency words, with a maximum Zipf value of 6.22 (i.e., Zipf values of 6 and 7 correspond to approximately 1000 and 10,000 occurrences per million, respectively; see van Heuven et al., 2014). For the description of the first-syllable frequency data we only have the B-Pal [BuscaPalabras] database (Davis & Perea, 2005) as reference. B-Pal calculates syllable frequency values for two- and three-syllable words based on the LEXESP database of Sebastián et al. (2000), a corpus of approximately five million Spanish words. As our syllable frequency calculations are based on a much larger corpus (170 million; see ‘Materials’ section for details), direct comparisons are not appropriate, but correlations between values for the shared words are. The paired correlations showed that our type and token syllable frequency calculations are both highly matched to those from B-Pal, with *r* = .944 for the type and *r* = 0.900 for the token frequencies (*N* = 2143 and *p* < .001 for both correlations). Overall, the words in the data set seem to be low-motor-content, low-to-mid-frequency, late-acquired, and with mid-values for length and neighbourhood.

**Table 1:** Descriptive statistics of the mean RT and the other variables

	N	Mean	S.D.	Median	Min	Max
Response times (RT)	4562	526.61	37.36	522.76	415.07	729.69
Motor content (MC)	4562	2.95	1.03	2.81	1.13	6.69
Age of acquisition (AoA)	4562	9.87	2.61	10.14	3.14	16.23
Frequency (Zipf)	4469	2.77	0.93	2.78	0.51	6.22
Length – Letters (Let)	4562	8.48	2.00	8	2	17
Length – Syllables (Syl)	4562	3.37	0.87	3	1	7
Neighbourhood – Orthographic (OrN)	4469	3.18	2.75	3	0	25
Neighbourhood – Phonological (PnN)	4469	6.80	4.95	6	0	50
1st-Syllable Frequency – Type (FSF_TP)	4562	12,216.62	14,298.18	8016	2	46,938
1st-Syllable Frequency – Token (FSF_TK)	4562	367,574.54	431,620.20	222,227	30	1,400,161

*Note*: Frequency and neighbourhood values for 93 verbs were not available in the EsPal database

### Preparation of predictor variables and RT

First, we transformed both neighbourhood and syllable frequency measures to reduce their extreme positive skewness (all > 1.5). After the calculation of the log10(x+1) of the scores for these measures, the skew was 0.005 for the orthographic neighbourhood, −0.634 for the phonological neighbourhood, −0.789 for the type syllable frequency, and −0.844 for token syllable frequency. Next, we examined the potential problem of multicollinearity. The problem is that the overlapped information associated with predictors would distort the unique contributions to outcome variance of the given predictors, or it would show unstable effects between different samples (Cohen et al., 2003). High pairwise correlations indicate a potential problem of multicollinearity in a linear model or even in LMM. Therefore, we calculated the pairwise correlations between the psycholinguistic variables for the word set (see Table 2). We obtained correlations *r* ≥ .8, which is a commonly used threshold to suspect a collinearity problem (Cohen et al., 2003), for three pairs of variables: those for word length (number of letters and of syllables), those for neighbourhood size (orthographic and phonological), and those for syllable frequency (type and token). For the rest of the pairwise correlations, *r* ≤ .62.

**Table 2:** Correlation matrix (pairwise selection)

	1	2	3	4	5	6	7	8	9
1. RT	1.000								
2. MC	−.011	1.000							
3. AoA	.367***	−.066***	1.000						
4. Zipf	−.418***	−.100***	−.461***	1.000					
5. Let	.475***	−.113***	.238***	−.249***	1.000				
6. Syl	.427***	−.119***	.222***	−.261***	.863***	1.000			
7. LogOrN	−.399***	.041**	−.364***	.375***	−.567***	−.479***	1.000		
8. LogPhN	−.399***	.034*	−.372***	.499***	−.620***	−.558***	.889***	1.000	
9. LogFSF_TP	.035*	−.020	.023	−.009	.109***	.299***	−.026	−.030*	1.000
10. LogFSF_TK	.029*	−.040**	.020	.027	.127***	.303***	−.029	−.026	0.988***

*** *p* < .001, ** *p* < .01, * *p* < .05. *RT* response times, *MC* motor content, *AoA* age of acquisition, *Zipf* logarithmic transformation of word lexical frequency, log10(fpmw)+3; Let, word length in number of letters; Syl, word length in number of syllables; LogOrN, log transformation of word orthographic neighbourhood, log10(OrN+1); LogPhN, log transformation of word phonological neighbourhood, log10(PhN+1); LogFSF_TP, log transformation of type first-syllable frequency, log10(FSF_TP); LogFSF_TK, log transformation of token first-syllable frequency, log10(FSF_TK)

In order to avoid the potential problem of multicollinearity, we selected only one variable of each highly correlated pair as predictor variable in the LMM analyses. We selected in each pair the variable with the highest correlation with the naming time and, subsequently, with the highest variability in terms of range and standard deviation. Therefore, we selected the number of letters, the (log-transformed) phonological neighbourhood size (*M* = 0.80, *SD* = 0.27, min = 0.00, max = 1.70), and the (log-transformed) type first-syllable frequency (*M* = 3.71, *SD* = 0.70, min = 0.30, max = 4.67), and we dropped the number of syllables, the (log-transformed) orthographic neighbourhood (*M* = 0.54, *SD* = 0.26, min = 0.00, max = 1.41), and the (log-transformed) token first-syllable frequency (*M* = 5.17, *SD* = 0.72, min = 1.47, max = 6.15) for further analyses. The next step was to standardize the selected predictors, because it is critical for the estimation of interaction effects. Moreover, transforming the predictors into the same scale allows a straightforward comparison of the effects. Finally, following common practice, we transformed RT to log10(RT) in order to reduce its skewness of 1.11 in the distribution of latencies, which resulted in 0.530 after transformation (*M* = 2.71, *SD* = 0.07, min = 2.42, max = 3.04). Then, we standardized the log-transformed latencies for a more intuitive interpretation of coefficients and plots, as the predictor variables were also standardized. We note that we repeated the same LMM analyses on the log-transformed and on the untransformed RT to check whether there was any difference in the results due to the log transformation. The results were the same in terms of the parameters that comprised the best-fit models achieved with transformed and non-transformed RT and in terms of the fixed effects that were statistically significant in the given models. Nevertheless, we chose to report here only the results of the analyses on the standardized log-transformed RT, for two reasons: (1) the conditional *R*^2^ (i.e., that due to random effects) was a bit higher, and (2) more adequate homoscedasticity and normality of residual distribution were found. All trial-level data are available as supplementary materials to this paper for readers who may wish to replicate our analyses or perform new ones.

### Initial phoneme characteristics and word stress

In order to capture the variance associated with voice key biases and with stress patterns of words, we dichotomously (1 = presence; 0 = absence) coded the phonetic characteristics of word onsets into 13 categories (i.e., vowel, alveolar, bilabial, dental, fricative, labiodental, interdental, liquid, nasal, palatal, occlusive, velar, and voiced), following a commonly used scheme (Balota et al., 2004); we also coded the stress patterns into two categories (i.e., paroxytone and oxytone classes). As some of the 15 resulting categories were highly correlated, and such a number of variables would likely cause problems of multicollinearity and convergence problems in the LMM analyses, we reduced the given factors using multiple correspondence analysis (MCA) (package *FactoMineR* 2.4., Lê et al., 2008). The factor loadings, in terms of squared cosine (cos^2^), are reported in Table 3. The results showed eight dimensions each accounting for more than 5% of the variance and together for about 90% of the total variance among word onset and stress predictors. The dimensions were labelled Dim_x, where ‘x’ = 1–8. The dimensions relate to the following: for Dim_1, alveolar, liquid, and voiced features; Dim_2, vowel and occlusive features; Dim_3, oxytone and proparoxytone stresses^2^; Dim_4, fricative feature; Dim_5, bilabial and nasal features; Dim_6, dental and velar features; Dim_7, labiodental and interdental; and Dim_8, palatal. Then, phonetic onsets and stress patterns were dichotomously (1 = member; 0 = no member) recoded into the corresponding dimension. Finally, it must be noted that these dimensions and their effects on the model are not of theoretical interest in this paper, and therefore we enter them together first in the LMM analyses only to control for the phonetic onset and stress biases on naming times.

**Table 3:** Multiple correspondence analysis of the 15 onset and stress features of words

Feature	Dim_1	Dim_2	Dim_3	Dim_4	Dim_5	Dim_6	Dim_7	Dim_8
Alveolar	.68							
Liquid	.45			.38				
Voiced	.54							
Vowel		.65						
Occlusive		.85						
Oxytone			1.00					
Paroxytone			1.00					
Fricative	.34			.49				
Bilabial					.66			
Nasal					.64			
Dental						.49		
Velar						.53		
Labiodental				.32			.47	
Interdental							.39	.34
Palatal								.66

*Note*: Cos^2^ scores lower than .30 are not shown

### Model construction

We strove to follow the best practice guidance from the seminal paper by Meteyard and Davies (2020) concerning the use of LMM in psychology. Most of the decisions we made regarding the rationale of the analysis process and results reporting are based on the advice given in that guideline. We agree that reporting results from LMM analyses in a comprehensive and systematic way, such as the one proposed by Meteyard and Davies (2020), may improve the understanding and transparency of the psycholinguistic findings based on that technique.

We examined the standardized log-transformed latencies of the correct responses to the verbs in the naming task fitting several LMM to estimate effects using the *lme4* package version 1.1-26 (Bates et al., 2015, 2020) in R version 4.0.4 (R core team, 2021), via RStudio version 1.4.1106 (RStudio Team, 2021). The construction rationale of the model was a minimal to maximal-that-improves-fit process to respond several research questions. To that end, we first selected a basic random-effects structure and built a baseline model. Then, we added the relevant predictor variable/s to answer the research questions presented in the introduction:

1. Do word frequency, AoA, length neighbourhood size, and/or syllable frequency affect verb naming latencies?
2. Does motor content of words influence verb naming latencies beyond the former variables?
3. Does motor content interact with any of the above-mentioned psycholinguistic variables?
4. Do AoA and frequency interact?

In the construction process, we examined whether the addition of each fixed effect to the model was justified by improved model fit to data. We evaluated model fit using likelihood ratio test (LRT, see, e.g., Baayen et al., 2008) comparisons because this index is recommended for testing nested data and is guided by research question models. However, the comparisons were also complemented with the Bayesian information criterion (BIC) and the Akaike information criterion (AIC) (e.g., Burnham & Anderson, 2016), as recommended by Meteyard and Davies (2020). We answered each of our research questions consecutively, examining whether the addition of a fixed effect of interest improved model fit. The structure of the reported results follows the same rationale. After addressing all the research questions, the model best fitted to the data was thoroughly examined. For the *final* model, we report all parameter estimates for each fixed effect (i.e., coefficients, standard errors, confidence intervals, *t*-tests, and *p*-values derived from the *lmerTest* package, which employs Satterthwaite approximations to denominator degrees of freedom; Kuznetsova et al., 2017), standard deviations, and variance estimates for each random effect (*lme4* package), three measures of the model quality and goodness of fit (i.e., marginal and conditional *R*^2^, intraclass correlation coefficient [ICC] for the random effects, and the root mean squared error, RMSE; all derived from the *performance* package [Lüdecke et al., 2020]), and the higher variance inflation factor (VIF) found for a term as an indicator of the magnitude of multicollinearity (derived from the *performance* package). We also present some plots of predicted values for the significant terms in the final model.

As we noted in the ‘Materials’ section and in Table 1, 93 verbs were not present in the EsPal database, and therefore they do not have values for word frequency and neighbourhood size. We had to remove the observations corresponding to those 93 items for the following LMM analyses because model fit comparisons need to be based on the models obtained from the same observations, such that the data set can have no missing data for the variables of interest. After that, the data pool comprised 98,447 observations.

### Baseline model: Random-effects intercepts

As our research questions concerned the effects of the selected variables (motor content, syllable frequency, word frequency, AoA, length, and neighbourhood size), we considered as a baseline model the one only composed of random effects. The specification of the appropriate random-effects structure is not trivial. The so-called *maximal* model (Barr et al., 2013) includes the full structure of random effects justified by the design (intercepts and slopes of variables of interest) in order to reduce type I errors (i.e., rejection of the null hypothesis when it is actually true), but this can easily result in convergence issues, in overparameterized and, likely, uninterpretable models (see Bates et al., 2018), or even in loss of power (Matuschek et al., 2017). We selected a basic random-effects structure according to our experimental design, trying to balance statistical power for testing the research questions and type I error. First, we included the between-participants and between-items effects in the model. This means that only the variance due to unexplained differences between sampled participants and words in random intercepts was considered. This simple model was run and converged, showing that it fit to the data (see model ‘RE_PI’ in Table 4). Then, we included the variable *block*. Block is a categorical variable that gathers the 10 different sets of the items used in the experiment. The blocks we used constituted the random selection of items from the complete set of 4562 verbs, but we must consider that billions of other possible combinations of items grouped into blocks could have been obtained from the same pool. The variable block in a way represents a series of 10 equivalent experiments done with different samples of items. Therefore, if we want to generalize our results to all other possible random samplings of *N* items, where *N* is the same number of items per block we used, the variance due to unexplained differences between blocks (random intercepts) must be separately reflected in the model. The model including the random intercept of blocks also converged and showed a better fit to the data than the previous one, with higher log-likelihood (LL) and lower AIC and BIC values. The LRT comparison was significant (see model ‘RE_PIB’ in Table 4). We decided not to include more random effects at this point of the construction process in order to cope with the tests for fixed effects without loss of power. However, we will contrast the random effects of slopes and associated correlations *after* addressing the fixed effects of interest. Adding more random effects to the final model allows us to check (1) whether there are improvements in the measures of model quality and in the goodness of fit, and (2) whether a more complete random-effects structure slightly affects the parameter estimates for each fixed effect of interest, especially in their coefficients, *t*-tests, and *p*-values (see Barr et al., 2013; Brauer & Curtin, 2018). Thus, the model ‘RE_PIB’ was considered as the baseline model, on which we add the variable/s of interest, as fixed effects, to address each research question. If the added fixed-effects terms improve model fit, that new model is used as the reference to check the subsequent terms added and so on.

**Table 4:** Model-building process and comparisons

Model specification	Model name	Nested model	FE added	Random effects			Model fit				LRT vs nested model	
				Participants	Items	Blocks	AIC	BIC	LL	*df*	*df*	χ^2^
RE only	RE_PI	-	-	Intercept	Intercept	-	221317	221355	−110654	4		
RE only	RE_PIB (bl)	RE_PI	-	“	“	Intercept	220993	221041	−110492	5	1	325.6***
FE_onset	FE_O	bl	Dim_1-to-8	“	“	“	220235	220349	−110106	12	7	771.8***
FE Main effects	FE_L	FE_O	Letters	“	“	“	218614	218737	−109294	13	1	1623.8 ***
FE Main effects	FE_LF	FE_L	Zipf	“	“	“	217695	217828	−108834	14	1	920.2***
FE Main effects	FE_LFN	FE_LF	LogPhN	“	“	“	217627	217769	−108798	15	1	70.7***
FE Main effects	FE_LFNA	FE_LFN	AoA	“	“	“	217387	217539	−108677	16	1	241.7***
FE Main effects	FE_LFNAS	FE_LFNA	LogFSF_TP	“	“	“	217385	217546	−108675	17	1	4.128*
FE Main effects	FE_LFNASM	FE_LFNAS	MC	“	“	“	217386	217556	−108675	18	1	1.352
Two-way interactions	FE_LFNAS _2W_M:L	“	MC:Let	“	“	“	217386	217557	−108675	18	1	0.991
Two-way interactions	FE_LFNAS _2W_M:F	“	MC:Zipf	“	“	“	217386	217557	−108675	18	1	0.772
Two-way interactions	FE_LFNAS_2W_M:N	“	MC:LogPhN	“	“	“	217387	217558	−108675	18	1	0.118
Two-way interactions	FE_LFNAS_2W_M:A	“	MC:AoA	“	“	“	217378	217549	−108671	18	1	9.059**
Two-way interactions	FE_LFNAS_2W_M:A_M:S	FE_LFNAS_2W_M:A	MC:LogFSF_TP	“	“	“	217379	217560	−108671	19	1	0.564
Two-way interactions	FE_LFNAS_2W_M:A_F:A (final)	“	Zipf:AoA	“	“	“	217362	217543	−108662	19	1	17.57***
Two-way interactions	FE_LFNAS_2W_M:A_F:A_F:S	final	Zipf:LogFSF_TP	“	“	“	217362	217552	−108661	20	1	2.364
Two-way interactions	FE_LFNAS_2W_M:A_F:A_L:S	“	Let:LogFSF_TP	“	“	“	217363	217553	−108661	20	1	1.587
Tuned RE-structure	Final_max	“	-	Intercept + slopes for Let + LogPhN + AoA + Zipf + LogFSF_TP+ MC:AoA + Zipf:AoA	“	“	215419	215932	−107656	54	35	2013.2***

*RE* random effects, *FE* fixed effects, *AIC* Akaike information criterion, *BIC* Bayesian information criterion, *LL* log-likelihood, *df* degrees of freedom, *LRT* likelihood ratio test, χ^2^, Chi-square, (bl), baseline. *** *p* < .001, ** *p* < .01, * *p* < .05. All models converged with no warnings except for the *final_max* model (the *bobyqa* optimizer of convergence was used). Sampling units: *N* total observations = 98,447, *N* participants = 126; *N* items = 4469; *N* blocks = 10

### Control of the phonological onset and stress effects

All the dimensions referring to the phonetic onset and stress of the words (except Dim_3, which was a constant) were introduced simultaneously in the model, since we were interested in these factors only as a methodological control of the possible biases caused by such factors. Seven terms for the main fixed effects of the dimensions were added into the model and its fit compared to that of the ‘baseline’ model. The new model converged, and the LRT comparison showed that the new terms for onset stress dimensions improved model fit substantially (see ‘FE_onset’ in Table 4).

### Tests of the main effects of predictor variables (research questions 1 and 2)

To test research questions 1 (i.e., the effects of syllable frequency, word frequency, AoA, length, and/or neighbourhood size on naming times) and 2 (i.e., the effects of motor content on naming times), we added in the model the terms corresponding to the main effects of variables. We determined the order of inclusion of each term on the basis of the pairwise correlation strengths between naming times and each variable, with the most highly correlated variable going first. The order was word length, word frequency, phonological neighbourhood, AoA, type syllable frequency, and finally motor content. Thus, the term for the main effect of number of letters was added in the model and its fit compared to that of the ‘FE_onset’ model. The new model converged, and the LRT comparison was significant, showing that the term for word length improved the model fit (see model ‘FE_L’ in Table 4). We followed the same procedure with the rest of the variables. The main effects of word frequency, neighbourhood, AoA, and syllable frequency separately and cumulatively improved model fit, and each LRT was significant (see models ‘FE_LF’, ‘FE_LFN’, ‘FE_LFNA’, and ‘FE_LFNAS’, respectively). Although the results of these tests suggest that word frequency, AoA, length, neighbourhood size, and syllable frequency affect naming latencies, we should wait until the examination of the final model to check whether the effects of these variables, as well as their directions and coefficients, remain statistically significant. By contrast, adding the term for the main effect of motor content did not improve any model fit indicator, and the LRT was not significant (see ‘FE_LFNASM’). According to the modelling process, we dropped the model that included the term for motor content and retrieved the previous one (i.e., ‘FE_LFNAS’) as a reference model for the next analysis.

### Test of the interactions between the motor content and the remaining predictor variables (research question 3)

We performed new tests to contrast the effects of all possible two-way interactions between the effect of motor content and the effect of each of the remaining psycholinguistic variables. The initial reference model was now the best-fit model so far (i.e., ‘FE_LFNAS’), which describes the main effects of word frequency, AoA, length, neighbourhood size, and syllable frequency. We added the effect of the interaction of motor content and length (MC:Let), and the LRT comparison indicated that the new term did not improve model fit (see ‘FE_LFNAS_2W_M:L’ in Table 4). Following the same procedure, we added separately the interaction terms for motor content and word frequency (MC:Zipf), motor content and neighbourhood size (MC:LogPhN), motor content and AoA (MC:AoA), and finally motor content and syllable frequency (MC: LogFSF_TP) . As presented in Table 4 (see models tagged ‘FE_LFNAS_2W_M:F’, ‘FE_LFNAS_2W_M:N’, ‘FE_LFNAS_2W_M:A’, and ‘FE_LFNAS_2W_M:A_M:S’), only the penultimate term added (i.e., MC:AoA) showed a significant LRT and better AIC, BIC, and LL scores of model fit than the contrast model (i.e., ‘FE_LFNAS’). Therefore, the model best fitted to the data so far was the one comprising the main effects of word frequency, AoA, length, neighbourhood size, and syllable frequency *plus* the effect of the interaction between motor content and AoA (model ‘FE_LFNAS_2W_M:A’).

### Test of the interaction between AoA and frequency (research question 4)

We performed similar two-way interaction tests to address whether the interaction between word frequency and AoA affect verb naming latencies. We added the term of the given interaction (Zipf:AoA) to the rest of the parameters of the model ‘FE_LFNAS_2W_M:A’ and contrasted their model fits. The LRT comparison was significant, showing that the included interaction term improved model fit (see model ‘FE_LFNAS_2W_M:A_F:A’ in Table 4).

### Final regression model

Once all research question were addressed, we considered the model comprising the main effects of word frequency, AoA, length, neighbourhood size, and syllable frequency *plus* the effect of the interaction between motor content and AoA, and *plus* the effect of the interaction between word frequency and AoA (i.e., model FE_LFNAS_2W_M:A_F:A) as the model best fitted to the data or, putting it more simply, the ‘final’ model. A comprehensive detailed description of the model is given in Table 5 following the Meteyard and Davies’ (2020) guidelines. At the bottom of the table we report three measures of model quality and goodness of fit. The obtained conditional *R*^2^ reveals that both the fixed and random effects of the final model explain 52.7% of the variance. Marginal *R*^2^ means that the proportion of variance explained by the fixed effects alone is 7.3%. These proportions indicate that the greatest proportion of variance in the data is explained by the differences between participants, items, and blocks. Similarly, the observed adjusted ICC (i.e., when taking all sources of all random effects into account) means that the proportion of the total variance in response times that is accounted for by the random factors is 48.9%. In other words, it means that including the random factors in the model makes sense. The RMSE will be used to evaluate model fit when we tune the random-effects structure up in the next section. Moreover, the final model showed no signs of multicollinearity, because the highest VIF was 2.44, which corresponded to the term of onset dimension 4 (Dim_4).

**Table 5:** Final model description

Fixed effects					
	Coefficients	*SE*	95% CI	*t*	*p*
Intercept	0.2048	0.0713	0.0643 / 0.3453	2.873	.00519
Dim_1	−0.1415	0.0115	−0.1641 / −0.1189	−12.251	2×10^−16^
Dim_2	−0.0885	0.0111	−0.1103 / −0.0668	−7.970	2×10^−15^
Dim_4	−0.5867	0.0188	−0.6236 / −0.5498	−31.122	2×10^−16^
Dim_5	0.0335	0.0129	0.0082 / 0.0589	2.590	.00962
Dim_6	−0.0002	0.0110	−0.0217 / 0.0212	−0.026	.97961
Dim_7	0.2630	0.0266	0.2110 / 0.3151	9.889	2×10^−16^
Dim_8	0.5606	0.0431	0.4763 / 0.645	13.006	2×10^−16^
Let_z	0.1400	0.0050	0.1303 / 0.1497	28.161	2×10^−16^
Zipf_z	−0.0898	0.0047	−0.0991 / −0.0806	−19.062	2×10^−16^
LogPhN_z	−0.0356	0.0060	−0.0473 / −0.0239	−5.942	3.03×10^−9^
AoA_z	0.0695	0.0043	0.0610 / 0.0780	16.017	2×10^−16^
LogFSF_TP_z	−0.0084	0.0044	−0.0171 / 0.0003	−1.897	.05790
MC_z:AoA_z	−0.0139	0.0043	−0.0192 / −0.0044	−3.213	.00132
Zipf_z:AoA_z	−0.0176	0.0440	−0.0234 / −0.0085	−4.015	6.03×10^−5^
Random effects					
	Variance		*SD*	ICC	
Participants (intercept)	0.4308		0.6563	.436	
Items (intercept)	0.0387		0.1968	.039	
Blocks (intercept)	0.0139		0.1180	.014	
Model fit					
Marginal *R*^2^	Conditional *R*^2^	Adjusted ICC	Conditional ICC		RMSE
.073	.527	.489	.453		0.700

*SE* standard errors, *CI* confidence intervals; Dim_1-to-8, onset and stress dimensions; Let_z, standardized scores of word length in number of letters; Zipf_z, standardized scores of logarithmic transformation of word lexical frequency, log10(fpmw)+3; LogPhN_z, standardized scores of log transformation of word phonological neighbourhood, log10(PhN+1); AoA_z, standardized scores of word age of acquisition; LogFSF_TP_z, standardized scores of log transformation of type first-syllable frequency, log10(FSF_TP); MC_z:AoA_z, interaction between standardized scores of motor content and AoA_z; Zipf:AoA, interaction between Zipf_z and AoA_z; RTlog10_z, standardized values of logarithmic transformation of response times, log10(RT). Confidence intervals, *t*-tests, and *p*-values are based on Satterthwaites’s methods for degrees of freedom and *t*-statistics. ICCs are based on the proposals by Nakagawa et al. (2017). Model equation, [RTlog10_z ~ Dim_1 + Dim_2 + Dim_4 + Dim_5 + Dim_6 + Dim_7 + Dim_8 + Let_z + Zipf_z + LogPhN_z + AoA_z + logFSP_TP_z + logFSF_TP_z + MC_z:AoA_z + Zipf_z:AoA_z + (1 | participants) + (1 | items) + (1 | blocks)]

**Fig. 2:**
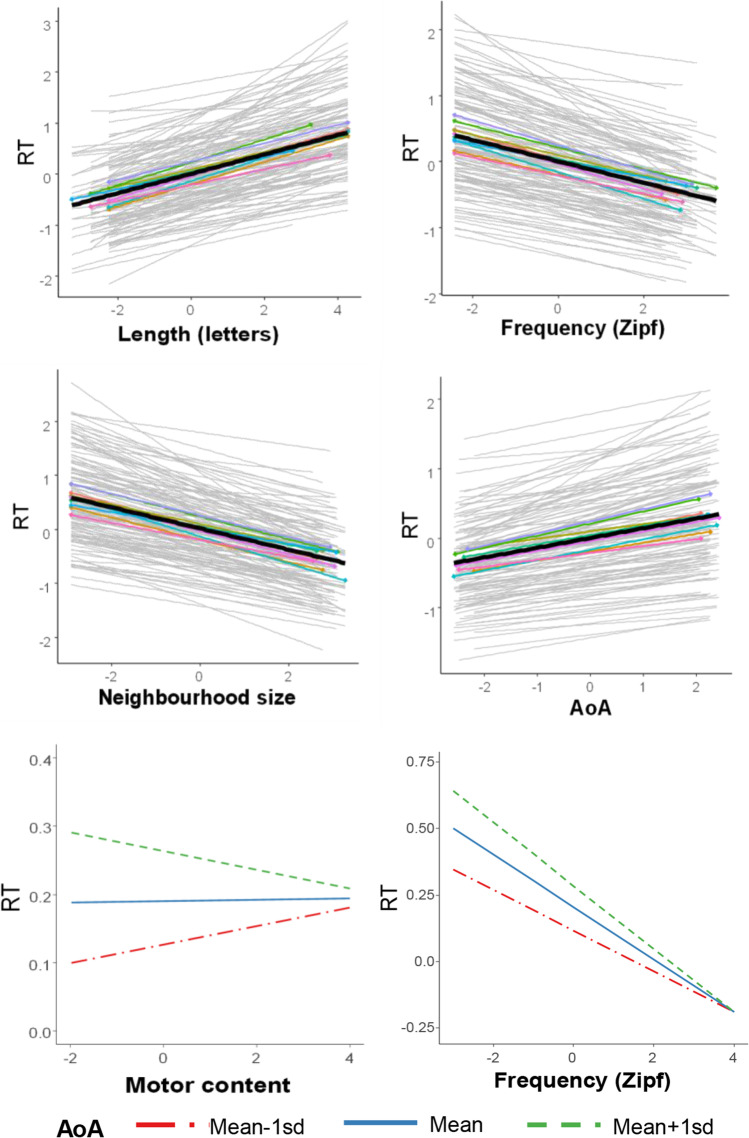
Regression charts of predicted values by fixed-effect terms (only significant psycholinguistic variables) of the final model and spaghetti plots of observed performance by participants and blocks of items *Note*: Main effect charts (first four plots, from top to bottom and left to right), predicted values in bold back line, block slopes in thin coloured (only for the electronic version of the article) dotted lines, and participant slopes in thin grey lines. Interaction effects charts (the two plots below), line styles represent three values of AoA: the mean, the value one standard deviation above, and the value one standard deviation below the mean (see legend). RT = standardized values of logarithmic transformation of response times, log10(RT)

Focusing now on the random-factor variances and their ICC (see Table 5), we can observe that each factor does not contribute equally to the explained variance, with individual variation (i.e., fast and slow participants) as the main source of the explained variance. This is visually shown by the dispersion of the grey lines along the RT axis at the spaghetti plots in Fig. 2, in which each line represents one participant’s performance. The very low variances and ICC of items and blocks as random effects indicate that these factors unrepresentative of the data (i.e., the low dispersion of observations by blocks can be visualized in the stacked coloured lines shown in the spaghetti plots, Fig. 2).

Regarding the fixed effects included in the model, we report the parameter estimates for each of them (i.e., coefficients, standard errors, confidence intervals, *t*-tests and *p*-values) at the top of Table 5. By inspecting coefficients and confidence intervals, and also the regression plots of Fig. 2, we can assess the impact of each fixed effect on verb naming times. Focusing on the effects of the psycholinguistics variables, the response speed slows with increasing word length and AoA, and with decreasing word frequency and neighbourhood size^3^, but the interpretation of the main effects of AoA and frequency are conditioned by the interaction terms. The regression chart of the interaction between motor content and AoA (bottom left plot, Fig. 2) shows how the weak but evident AoA effect on naming times for verbs with low ratings in motor content gradually decreases as motor content increases. Furthermore, we can observe that there is a positive slope of motor content at low AoA, a flat slope at mid-AoA, and a negative slope at high AoA scores. Regarding the interaction between word frequency and AoA, the regression chart (bottom right plot, Fig. 2) shows that the negative effect of frequency on RT increases with increasing AoA. Moreover, the AoA effect for low-frequency verbs gradually decreases as frequency increases and finally disappears at top-frequency scores. Finally, the syllable frequency effect was surprisingly not significant. As this was an unexpected result, we extended our research plan to explore possible explanations for this null effect. We explored whether the interactions between syllable frequency and word frequency and between syllable frequency and word length affect verb naming latencies. The interaction between syllable frequency and word frequency was introduced to check its null effect on word naming, which has been consistently reported across languages (e.g., Carreiras et al., 2006; Conrad et al., 2006; Macizo & Van Petten, 2006). The interaction between syllable frequency and word length was nevertheless introduced to explore a potential explanation of the main null effect of syllable frequency. As experiments reporting effects of syllable frequency, at least in Spanish, have usually used disyllabic and trisyllabic words (e.g., Carreiras & Perea, 2004; Dominguez et al., 1997), this interaction may show whether the syllable frequency effect in our experiment is modulated by the length of words, with a more apparent effect of syllable frequency in shorter (two-syllable) or mid-length (three-syllable) words than in longer words. Following the same procedure as the former analyses, we first added the term of the interaction between syllable frequency and word frequency (Zipf: LogFSF_TP) to the remainder of the parameters of the final model and contrasted their model fits. The LRT comparison result was not significant. The same was done with the interaction between syllable frequency and word length (Let: LogFSF_TP), and the same result was obtained (see models ‘FE_LFNAS_2W_M:A_F:A_F:S’ and ‘FE_LFNAS_2W_M:A_F:A_L:S’ in Table 4, respectively). Consequently, the final model described above was not modified by these additional tests.

Although the final model gathers all fixed effects of interest, it still needs to be checked on its random-effects structure in order to ensure that the fixed effects found and reported so far are reliable and not due to an excessive type I error rate.

### Tuning the final model to a maximal random-effects structure

The models reported so far have incorporated fixed effects due to the psycholinguistic variables of interest and random effects due to the differences between participants, items, and blocks in intercepts. Not including random slopes of the within-participants, within-items, or within-blocks effects of interest could increase the probability of finding a fixed effect as statistically significant when it actually is not (i.e., type I error). However, the counterpart of including so many random effects (i.e., intercepts, all slopes, and their correlations) is that it could yield a loss of sensitivity and/or an overparameterization of the model, making it senseless. The strategy to deal with this omission was to use the final model as reference model and add the same fixed-effect terms as correlated slopes in the random-effects structure (i.e., the maximal model proposed by Barr et al., 2013). In our case, all fixed-effects terms included in the final model correspond to the phonological onset dimensions and the psycholinguistic variables of interest, which vary within participants (i.e., each participant responded to the items with different phonological onsets and psycholinguistic features) and between items (i.e., items differ from each other in their onsets and in their psycholinguistic features). However, as the onset dimensions are not of interest to us and they may generate an overparameterization of the model and, as a consequence, convergence issues, we ‘maximized’ the model only with the random slopes (and their correlations) of the psycholinguistic variables of interest into the random effect of participants. If the maximal model converges, it will be contrasted with the final model in order to check whether model fit improves and, more importantly, whether the given maximal structure critically affects the coefficients of the fixed effects of interest. According to Barr et al.’s (2013) simulations, a maximal random-effects structure would correct the parameter estimates for each fixed effect and keep the type I error rate at 5%. We ran the maximal model; it converged (although with a warning of singular fit) and improved model fit, with much better AIC, BIC, and LL scores than those for the non-maximal final model (see *final_max* model at the bottom of Table 4). A detailed description of the final_max model is given in Table 6. The measures of the quality of the model show that the final_max model is better fitted than the final model. The marginal *R*^2^ of the final_max model is the same as that measured in the final model (.073), although the conditional *R*^2^ is a bit higher for the final_max model than for the final model (.539 vs .527, respectively), which indicates an increase only for the variance explained by the random effects in the final_max. Moreover, the higher ICC and the lower RMSE scores in the final_max model than those in the final model evidence better quality of the former model (see Table 6). Regarding multicollinearity, the final_max model showed low VIF scores, with the highest being 2.33 for the term of onset dimension 4 (Dim_4) parameter.

**Table 6:** Final_max model description

Fixed effects									
	Coefficients	*SE*		*t*			*p*		
Intercept	−0.2045	0.0713		2.866			.0052		
Dim_1	−0.1421	0.0115		−12.341			2×10^−16^		
Dim_2	−0.0859	0.0110		−7.768			9.9×10^−15^		
Dim_4	−0.5844	0.0188		−31.091			2×10^−16^		
Dim_5	0.0333	0.0129		2.585			.0098		
Dim_6	−0.0014	0.0109		−0.131			0.8957		
Dim_7	0.2624	0.0265		9.892			2×10^−16^		
Dim_8	0.5701	0.0429		13.265			2×10^−16^		
Let_z	0.1406	0.0083		16.890			2×10^−16^		
Zipf_z	−0.0885	0.0062		−14.333			2×10^−16^		
PhN_z	−0.0229	0.0068		−4.946			1.1×10^−6^		
AoA_z	0.0648	0.0064		10.130			2×10^−16^		
LogFSF_TP_z	0.0103	0.0055		−1.874			.0617		
MC_z:AoA_z	−0.0119	0.0038		−3.111			.0019		
Zipf_z:AoA_z	−0.0157	0.0041		−3.793			.0002		
Random effects									
	Variance	*SD*	Correlations						
			Interc.	Let_z	Zipf_z	LogPhN_z	AoA_z	LogFSF_TP_z	MC_z:AoA_z
Participants (intercept)	0.4331	0.6580							
Let_z (slope) | Participants	0.0055	0.074	−.08						
Zipf_z (slope) | Participants	0.0019	0.0444	−.26	−.29					
LogPhN_z (slope) | Participants	0.0013	0.0365	−.38	−.26	.13				
AoA_z (slope) | Participants	0.0019	0.0444	.66	.30	−.72	−.52			
LogFSF_TP_z (slope) | Participants	0.0012	0.0353	.10	.11	−.30	−.16	.34		
MC_z:AoA_z (slope) | Participants	4.9×10^−5^	0.0070	−.32	−.45	−.18	.14	−.26	.11	
Zipf_z:AoA_z | Participants	3.3×10^−4^	0.0181	.20	−.44	.58	−.42	−.28	.54	.01
Items (intercept)	0.0389	0.1972							
Blocks (intercept)	0.0138	0.1177							
Model fit									
Marginal *R*^2^	Conditional *R*^2^	Adjusted ICC	Conditional ICC				RMSE		
.073	.539	.503	.466				0.689		

SE, standard errors; CI, confidence intervals; Let_z, standardized scores of word length in number of letters; Zipf_z, standardized scores of logarithmic transformation of word lexical frequency, log10(fpmw)+3; LogPhN_z, standardized scores of log transformation of word phonological neighbourhood, log10(PhN+1); AoA_z, standardized scores of word age of acquisition; LogFSF_TP_z, standardized scores of log transformation of type first-syllable frequency, log10(FSF_TP); MC_z:AoA_z, interaction between standardized scores of motor content and AoA_z; Zipf:AoA, interaction between Zipf_z and AoA_z; RTlog10_z, standardized values of logarithmic transformation of response times, log10(RT). *t*-tests and *p*-values are based on Satterthwaites’s methods for degrees of freedom and *t*-statistics. ICC are based on the proposals by Nakagawa et al. (2017). Model equation, [RTlog10_z ~ Dim_1 + Dim_2 + Dim_4 + Dim_5 + Dim_6 + Dim_7 + Dim_8 + Let_z + Zipf_z + LogPhN_z + AoA_z + logFSP_TP_z + logFSF_TP_z + MC_z:AoA_z + Zipf_z:AoA_z + (1 + Let_z + Zipf_z + LogPhN_z + AoA_z + logFSP_TP_z + logFSF_TP_z + MC_z:AoA_z + Zipf_z:AoA_z | participants) + (1 | items) + (1 | blocks)]

Focusing now on the random effects, we observe that the variances for the participant, item, and block intercepts of the final_max model are almost identical to the corresponding variances of the final model. Regarding the effects of the slopes within participants, all estimated variances are very low, with some of them equalling zero (e.g., the variance of the interaction MC_z:AoA_z slope was 0.000049), which, on the other hand, also could be causing the singular fit of the final_max model. Following the recommendations of how to cope with singular fit (e.g., Bates et al., 2018), we checked the dimensionality of the random-effects distribution of the final_max model using principal component analysis (PCA; *lme4* package), in order to determine whether the random-effects structure could be simplified. The results from the PCA showed that the variance was completely accounted for by eight factors: the first factor accounted for 97.49%, the second factor for 1.45%, the third factor for 0.506%, the fourth for 0.271%, the fifth for 0.226%, the sixth for 0.052%, the seventh for 0.003%, and the eighth for virtually 0% of variance. That is, the PCA shows that the random-effects structure can be explained at 100% by seven factors, and at 99.99% by six factors. If we apply the criterion by Bates et al. (2018) that the number of dimensions to be reflected in the maximal random-effects structure is the number of underlying factors from PCA that cumulatively account for 100% of the variance, we could drop only one or maybe two random-effects terms from our final_max model. As the maximal random-effects structure of the final_max model (eight terms) was closely adjusted to the underlying dimensions found by PCA (seven factors explaining 100% of variance), the model properly converged, and we achieved our objective of reliably and comprehensively answering a series of research questions, we did not proceed with further analyses.

Importantly, the coefficients of the fixed effects in the final_max model are practically the same as the corresponding coefficients in the final model, and they all remain statistically significant (see Table 6). Therefore, we can claim that the effects of interest found in the final model were not inflated by a poor random-effects structure that would have increased the type I error rate. The maximal random-effects structure improved the model fit but barely affected the fixed-effects terms of the final model.

## Discussion

The main objective of this megastudy was to provide a behavioural database of word reading accuracy and speed data from healthy adults for a wide range of Spanish verbs. We collected those behavioural data for a total of 4562 verbs in a word naming task and have calculated and added into our database a number of psycholinguistic variables taken from other sources, namely, word length, syllable frequency, phonological and orthographic neighbourhood, lexical frequency, AoA, and motor content. This data set, which we call ‘SpaVerb-WN’, represents the main result of the largest megastudy of word naming in Spanish so far, making it an important and useful methodological tool that contributes to the scientific progress in the field of psycholinguistics, and in particular to word reading in Spanish (see Balota et al., 2012; Keuleers & Balota, 2015; Kuperman, 2015, for the advantages of megastudies). The database is available as supplementary material to this article.

Another aim of this work was to investigate the extent to which a number of sublexical, lexical, and lexical-semantic variables are involved in the reading of Spanish verbs. Previous studies found significant effects of syllable frequency, word length, neighbourhood density, lexical frequency, and AoA on naming times, although the effects vary from one language to another. More specifically, the largest differences can be found between languages with opaque spelling and languages with transparent spelling. In this regard, once the phonological onset effects were controlled for and the type I error of fixed effects was reduced by the maximal random-effects structure, we found reliable effects of word frequency, length, neighbourhood size, and AoA on verb reading times, which increase with increasing word length and AoA, and with decreasing word frequency and neighbourhood size. However, the interpretation of the main effects of AoA and word frequency is conditioned by the significant interaction found between them, and the interpretation of the AoA effect also is contingent on its interaction with motor content. Additionally, the influence of syllable frequency was unexpectedly not significant.

### Word length effect

The strongest effect, in terms of standardized regression coefficients, was observed for word length (measured in letters), with less time devoted to the reading of short words as compared to long words. This result is in line with the findings for nouns in Spanish (e.g., Cuetos & Barbón, 2006; Davies et al., 2013; 20014). With respect to what cognitive processes are related to word length effect, the most conventional hypothesis claims that it reflects the serial phonological encoding of graphemes, indicating that words are being processed through a serial sublexical pathway. Nevertheless, this explanation does not deny that words also can activate a parallel lexical route that mitigates the word length effect under certain factors (see Barton et al., 2014 for a review). One of those factors is the transparency of language: linear and large word length effects observed in languages with consistent spelling systems are a consequence of the relevant role of the sublexical pathway in word naming. By contrast, in more opaque languages, the length effect is diminished and proceeds in a more complex way (i.e., U-shaped form), possibly as a result of the prevalence of lexical processing, which is unaffected by the number of letters, and to its interaction with the highly inconsistent grapheme–phoneme correspondence of the sublexical pathway. Our data fit to this conventional explanation because the word length effect is the most apparent effect we have found in the present megastudy on word naming in Spanish, a language with high consistency in grapheme–phoneme correspondence.

### Word frequency and AoA effects

We have observed that naming times increase with a decrease in word frequency and with an increase in AoA. These effects have similar coefficients, but they are lower as compared to that of word length. On the one hand, the frequency effect is consistent with that found in some studies on reading in Spanish (Davies et al., 2013, 2014) and in other transparent languages (e.g., Burani et al., 2007 in Italian; Raman et al., 2004 in Turkish), but it challenges the null effect of word frequency obtained by Cuetos and Barbón (2006). The word frequency effect found in the present study also suggests that there is a lexical access in reading aloud in Spanish. On the other hand, the effect of AoA that we obtained confirms the findings in Cuetos and Barbón (2006) and Davies et al. (2013) in Spanish (but see Davies et al., 2014). Our results also add to those of previous studies that reported AoA effects in word naming in other transparent languages, with these results being robust in Turkish (Raman, 2006, 2018) but mainly null in Italian (see Barca et al., 2002; Burani et al., 2007; Davies et al., 2014; Wilson et al., 2012, 2013, for null effects; but see Bates et al., 2001, for positive effects). Our finding of a main effect of AoA on verb naming in Spanish contradicts the prediction for transparent languages from one of the prominent explicative hypotheses on AoA effects, the *arbitrary mapping hypothesis* (Ellis & Lambon Ralph, 2000; Lambon Ralph & Ehsan, 2006; Monaghan & Ellis, 2002). This hypothesis claims that the AoA effect naturally emerges from any cognitive network that establishes arbitrary mappings between input (e.g., orthography) and output (e.g., phonology or semantics) representations. The information which is entered early in the network adjusts its configuration (i.e., the weights of the connections) to facilitate the learning of that information (and any other type of similar information), but this has a cost such that the network gradually reduces its plasticity to incorporate new unrelated mappings. Then, the information which is introduced later does not have the same capacity for adjusting the configuration as previously. As a result, this information is learned with less accuracy. This hypothesis predicts null or reduced AoA effects on word naming in languages with transparent orthographies, because the mappings shaped by early regular or predictable input-to-output patterns would favour the learning of late-entered regular patterns, which occurs in languages with highly predictable letters-into-sounds conversion. However, this is not supported by our results, which show almost the same size effects for AoA and for word frequency. Therefore, the main effect of AoA requires an additional discussion considering the two-way interaction effects between AoA and word frequency and between AoA and motor content.

### Interaction between frequency and AoA effect

We have found that the negative effect of frequency on RT increases with increasing AoA, and that the AoA effect for low-frequency verbs gradually decreases as frequency increases. According to our knowledge, the effect of the interaction of AoA and word frequency on word naming time has never been reported in a transparent language until now (see Burani et al., 2007; Wilson et al., 2013, for a null interaction effect in Italian and Spanish). However, the interaction mostly appears in English (e.g., Catling & Elsherif, 2020; Cortese & Schock, 2013; Cortese et al., 2018; Dirix & Duyck, 2017; but see Juhasz & Rayner, 2006). This interaction suggests that frequency and AoA may share their locus of action in one or more stages of word reading in a transparent language as well. Thus, if the cognitive mechanisms of word frequency are associated with the lexical retrieval stage during word recognition and production, the locus of action of AoA could also be at lexical retrieval. The link between the word AoA and frequency effects is not new, and we will discuss it later considering the other significant interaction we found.

### Interaction between AoA and motor content effect

This study is the first to find an interaction between AoA and motor content. Motor content is a measure of the semantic quality of a verb in terms of the amount of displacement or movement of the different parts of the body involved in the execution of the action referenced by the given verb (San Miguel Abella & González-Nosti, 2020). Then, the interaction between motor content and AoA, in which the AoA effect on naming times for verbs with low ratings in motor content (e.g., ‘aceptar’, to accept) gradually diminishes as motor content increases (e.g., ‘amasar’, to knead), may locate the AoA effect at a semantic level. Furthermore, the interaction also shows an inhibitory effect of the motor content for the earlier-acquired words that disappears for mid-age-acquired words and turns facilitatory for the later-acquired words. Thus, this suggests that the influence of motor content on lexical processing varies with the age at which verbs were acquired.

Taking the interactions between AoA and frequency and between AoA and motor content together, we can first infer that word AoA shows a double influence on word reading: one in a lexical retrieval/activation stage and the other at a semantic level. This double locus of action of AoA fits the explanation given by Brysbaert and Ghyselinck (2006). They analysed multitask investigations of word processing and observed that there is a frequency-related AoA effect, which is similar in magnitude to the frequency effect and is associated with tasks without semantic mediation, and another frequency-independent AoA effect mainly observed in tasks that require a semantic analysis of the input. This last AoA effect may be caused by the competition between different representations at the conceptual and/or lemma level, which in fact is the *semantic hypothesis* of the AoA (Brysbaert et al., 2000; Ghyselinck et al., 2004). According to this hypothesis, AoA affects how meanings are represented and the rules of the organization of the semantic system. Our effects of AoA and word frequency are consistent with the frequency-related AoA effects observed by Brysbaert and Ghyselinck (2006), who inferred that both effects are due to the same learning process. Moreover, the significant interaction between those variables allows us to add that both variables affect the same process, likely the lexical retrieval/access. However, Brysbaert and Ghyselinck’s theory does not predict frequency-independent AoA effects in the word naming task, but in tasks with semantic mediation, such as picture naming and word association, categorization, or generation. Overall, the word naming task does not involve semantic access, although this does not rule out that some words can be processed semantically depending on their characteristics (e.g., high-imageability words). For example, Davies et al. (2014) and Wilson et al. (2013) found an interaction between AoA and imageability such that, when reading words in Spanish, the AoA effect is evident for words that are likely to induce a semantic involvement, but not for those with low (or slow) semantic activation. Similarly, Raman (2018) observed that the AoA effect disappeared in word naming in Turkish when low-imageability or low-frequency fillers were introduced in the word set. She suggested that the development of reading strategies in word naming according to task demands is a universal process, and that the AoA effect diminishes as reading is less semantic, even in transparent languages. The interaction between AoA and motor content found in this study may be interpreted in a similar way. The embodiment theory argues that the semantic content related to actions is represented in a less abstract, more sensorial form, and therefore verbs that involve movements are associated more with sensorimotor neural networks and less with the conceptual-abstract ones. If so, we can infer from our interaction effect between AoA and motor content of verbs that the AoA effect is more apparent for those verbs that are more abstract (i.e., verbs with low motor content ratings) than for less abstract verbs (i.e., those with high motor content ratings). This would be the second locus of action inferred for the AoA effect observed in the present work, and it may correspond to the independent-frequency AoA effect proposed by Brysbaert and Ghyselinck (2006). This interpretation also relies on the results obtained by Davies et al. (2013) in another megastudy of word naming in Spanish. As the authors obtained high correlations between the predictor variables they studied, they performed PCA on those variables to derive orthogonalized predictors. The PCA located the AoA into two factors: one formed by word frequency, familiarity, and AoA, which was labelled the ‘frequency’ factor, and another ‘semantic’ factor comprising imageability, familiarity, and AoA. This distribution of AoA clearly matches the frequency-related and frequency-independent AoA effects suggested by Brysbaert and Ghyselinck (2006). Davies et al. (2014) found that both predictor factors were significant in explaining naming times.

As mentioned before, Brysbaert et al. (2000; also Ghyselinck et al., 2004) provided empirical support to the semantic hypothesis of the AoA. Additionally, Steyvers and Tenenbaum (2005) provided an explanation of why and how AoA influences the organization of the semantic system. They proposed a model of semantic growth where the order of learning affects the connectivity of the network. Early-learned concepts become more densely connected and centred in the network than late-learned concepts. As a consequence, there is a cognitive access bias toward highly connected or central nodes (i.e., early concepts) that, by default, are accessed sooner than those less connected or more dispersed (i.e., late concepts). Linking this explanation to the interpretation of the interaction between AoA and motor content, and assuming that high motor content of verbs reflects a more embodied, less abstract representation of actions, we can speculate about how the representation of actions changes during an individual’s lifetime and how this affects word processing. Earlier words were stored in a semantic network, likely as Steyvers and Tenenbaum (2005) explained, and that network may capture more abstract features and less information of the motor and sensory system. As time passes, motoric-sensorial information is also represented but in a different ‘semantic’ or maybe in a mixed network (i.e., with information partially in an abstract-semantic and in another embodied-semantic network). When word reading is required, only the abstract-semantic network, as part of the linguistic system, is consulted, so that only words associated with abstract-semantic features show a benefit in lexical tasks. Nevertheless, this is simply a speculative proposal to analyse the novel effect of motor content in verb naming, and more research is needed in this respect. On this matter, observing the AoA effect in people with some type of disease with motor impairment could be especially interesting and revealing.

### Neighbourhood size effect

We have found a significant facilitatory effect of the phonological neighbourhood, with words with larger neighbourhoods taking less time to read aloud. This matches the consistent effect found across languages in word naming with either orthographic or phonological neighbourhood size. A well-established explanation of the effect comes from PDP [pre-/during/post-] reading models, which claim that words with similar patterns strengthen their connections between orthography and phonology in such a way that they finally make up large neighbourhoods. Therefore, when activation of the phonological code is required, its recovery is facilitated by the number of neighbour words (Seidenberg & McClelland, 1989). The result we obtained for the phonological measure of neighbourhood size provides further evidence to previous studies which found that orthographic neighbourhood size affects reading in transparent orthographies (e.g., Burani et al., 2007; Davies et al., 2013). We also checked that the final regression model did not vary when using the phonological or the orthographic measure, which indicates that both measures play a similar role in word naming in Spanish. This may be because both neighbourhood measures in Spanish capture the concept of ‘phonographic’ neighbours (i.e., words differing in one letter and one phoneme, such as ‘stove’ and ‘stone’) better than other purely orthographic ways of calculating neighbourhood (e.g., ‘stove’ and ‘shove’). As Adelman and Brown (2007) showed, phonographic neighbours facilitate word naming, but not other purely orthographic neighbours.

### Syllable frequency (null) effect

The facilitatory effect of the initial-syllable frequency in word production has been observed extensively in word production tasks in Spanish and in other languages (e.g., Carreiras et al., 1993 in Spanish; Macizo & Van Petten, 2006 in English; Simpson & Kang, 2004 in Korean). This effect has been interpreted as a consequence of the role played by the syllable frequency at the phonetic encoding stage in speech production (e.g., Levelt et al., 1999). In this context, a facilitatory syllable frequency effect was expected in our megastudy of Spanish verbs, but surprisingly, no significant result was found in either the final or the ‘maximal’ model (i.e., the final_max model). We also found a null interaction between syllable frequency and word frequency, which has been consistently reported across languages (e.g., Carreiras et al., 2006; Conrad et al., 2006; Macizo & Van Petten, 2006). More critically, we found that the null effect of the syllable frequency is not modulated by length of words. In addition, we discard the possibility that the null effect of syllable frequency could be due to the chosen measure of type frequency instead of token frequency, for three reasons: both measures are very highly correlated (*r* = .988), type frequency is more correlated with naming times than token frequency, and previous evidence shows that type frequency explains the facilitatory effects found in naming better than token frequency (Conrad et al., 2008). Consequently, we could only advance a tentative explanation of the syllable frequency effect. Our materials are only composed of verbs, all of which have paroxytone or oxytone stress patterns, and only six of them have the stressed syllable in the initial position. By contrast, studies in Spanish that find a syllable frequency effect usually use two-syllable or three-syllable nouns, which are more likely to have the stressed syllable in the initial position. Therefore, it is possible that the syllable frequency effects emerge or are higher in words with the stressed first syllable, and consequently this was not detected in our set of verbs, where the stressed syllables are mostly in the last or next-to-last position. In any case, more research is needed in order to clarify the absence of this effect. Future investigations could, for instance, collect new behavioural data for a large set of nouns and check the syllable frequency effect; we would also consider promising a research programme that would specifically focus on the links between the effect of syllable frequency and those of other variables such as stressed syllable, bigram frequency, and second-syllable frequency, among others.

### Grammatical class

One objective of the present megastudy was to add evidence about whether the results obtained with nouns may be directly generalized to words of other grammatical categories, at least in word naming. On the one hand, results from Davies et al. (2013) suggest so. They compared the role of a number of psycholinguistic variables in word reading across three grammatical classes (i.e., nouns, verbs, and adjectives) and found no differences by grammatical class beyond the fact that verbs and nouns are read significantly more slowly than adjectives (see also Rodríguez-Ferreiro et al., 2009, for a null effect of grammatical class in a picture/action naming task). On the other hand, neuropsychological evidence (see introduction) suggests that other variables may be specifically involved in action/verb processing, which could apply to motor content. Overall, our results show no relevant differences in the role of the studied variables here in comparison to previous studies that employed nouns, except for the syllable frequency effect (which was discussed above). In addition, a significant effect was observed for the interaction of motor content and AoA of verbs with respect to the naming times of verbs, which could contribute to the knowledge of semantic networks of actions and suggest new hypotheses to examine in future studies.

### The robustness of the regression model

Finally, our results were obtained by means of a regression model in which we controlled for random intercepts by participants (and also the random slopes of the fixed-effects terms included in the model), by items, and by experimental blocks, and for the effects of the phonetic onsets of words as well. Moreover, the model converged despite its complexity because of the large number of observations we collected. Therefore, we consider that the last regression model we fitted (i.e., final_max model) reliably and comprehensively describes the involvement of the target psycholinguistic variables when reading verbs in Spanish.

## Limitations of the present work, and future research

There is a potential limitation of this study concerning the generalizability of the results to the general population, given that the data on accuracy and latencies were obtained from a sample of Spanish participants, with a relatively homogeneous educational level (university students), and where the great majority were women. Although Spanish is the native and official language of millions of speakers distributed across a large and diverse geographic and cultural area around the world, there are linguistic and cultural differences across the Spanish-speaking populations and communities. However, the above consideration only concerns a direct generalization of the present data to other populations. There is no reason to think that the results found here may be different from those of an equivalent megastudy carried out in other Spanish-speaking population with the appropriate psycholinguistic norms and data, since all variations of Spanish keep in common core features regarding spelling-to-sound correspondence, syllable limits, and accent rules. The biases of educational level and gender in our sample are commonly found in those of the vast majority of psychological studies. With respect to educational level, neither accuracy not RT data should be directly generalized to populations with other educational levels, since reading skills are linked to that factor. Moreover, the high educational level of our sample may be related with the ceiling effect we observed in accuracy. Regarding gender, there is no a priori reason to think that the overall results could be slightly different from a more balanced sample, although this assumption should be addressed empirically.

## Conclusions

Effects on word processing found in one language might not be directly transferable to others, especially between those that differ in the transparency of the spelling system. Under this premise, we developed a megastudy of word naming in Spanish, a transparent language, to provide an extensive behavioural database and to explore the role of phonetic, sublexical, lexical, and lexical-semantic variables. Moreover, we employed verbs because, up to now, there have been no studies carried out exclusively with verbs, so it was unknown to what extent results obtained with nouns may be directly generalized to words of other grammatical categories. In addition, verb production allows us to observe in particular the effect of motor content of actions, which may have an important impact on hypothesizing why some patients with motor deficits show difficulties in processing verbs with high motor content. The regression model obtained by LMM on naming times shows reliable effects of word frequency, length, neighbourhood size, and AoA, but not of syllable frequency. Interpretations of further interactions between AoA and word frequency and between AoA and motor content are in accord with the dual locus of action of AoA proposed by Brysbaert and Ghyselinck (2006). Overall, the results are not far from those previously found when reading nouns in Spanish, except for the syllable frequency effect. The findings also support the idea that multiple lexical and sublexical routes operate in parallel in word reading in transparent languages. Finally, this megastudy suggests a new and specific research area on motor content of verbs and their abstract/embodied representational status.

## Footnotes

1 We include in the analyses all responses registered in the rescue block. Therefore, some items received 41 responses instead of 20 or 21.

2 Dim_3 has the maximum load of each stress pattern, because it is in fact a constant, since all the words have an oxytone or proparoxytone stress. Therefore, Dim_3 was discarded for any further analysis.

3 In parallel to the main research plan, we tested possible differences in the regression model due to the different measures of neighbourhood (phonological vs orthographic). For that purpose, we calculated an alternative version of the final model to contrast with, where the measure of phonological neighbourhood was replaced by the orthographic one. The LRT comparison result was not significant (AIC = 217371, BIC = 217551, LL = −108666, χ^2^ = 0.0), showing that the orthographic neighbourhood does not improve model fit in relation to the equivalent including the phonological neighbourhood instead. Although the coefficient for the orthographic neighbourhood term was significant (*t* = −5.115, *p* <. 001), this was weaker than that of the phonological neighbourhood in the final model (−0.0261 and −0.0356, respectively).
